# Supplementary material for: Dietary Iodine Intake and Related Factors among Secondary School Students in Macao
Source: Healthcare (Basel). 2023 May 18;11(10):1472. doi: 10.3390/healthcare11101472 (PMC10218052; doi:10.3390/healthcare11101472)
Supplement: Supplementary file 1 [file healthcare-11-01472-s001.zip › healthcare-2286685-supplementary.pdf]

Manuscript ID: healthcare-2286685

Type of manuscript: Article

Title: Dietary iodine intake and related factors of secondary school students in Macao

Authors: Cleandy Lei, Xiao Yu Tao, Ying Xiao*

Table S1. Iodine intake of subjects from various food items.

Food item	Mean ± SD (μg)	Median (μg)
**Cereals and grains**	**2.1 ± 1.4**	**1.8**
Rice	0.9 ± 0.6	0.9
Congee	0 ± 0	0.1
Noodles	0.3 ± 0.3	0.2
Steamed bread	0.1 ± 0.5	0
Bread	0.7 ± 0.8	0.6
Biscuit	0 ± 0	0
**Starchy rhizomes**	**0.2 ± 0.4**	**0.2**
Potato	0.2 ± 0.4	0.2
**Soybeans products**	**2.8 ± 5.2**	**1.3**
Tofu	2.1 ± 4.5	1.1
Dried bean curd	0.2 ± 0.6	0
Soy milk	0.5 ± 1.0	0.2
**Vegetables and legumes**	**4.8 ± 4.0**	**4**
Dark-green leafy vegetables	2.3 ± 1.9	2.5
Light-green leafy vegetables	0 ± 0	0
Gourd	0.3 ± 0.5	0.1
Legume	0.4 ± 0.8	0.1
Carrot	1.7 ± 2.3	0.2
Tomato	0.2 ± 0.4	0.1
**Seaweed and mushrooms**	**15.7 ± 41.5**	**5.8**
Mushroom	2.7 ± 4.2	1.4
Kelp	6.1 ± 23.7	0
Dried seaweed	6.9 ± 20.4	1.2
**Fruits**	**1.5 ± 2.1**	**0.8**
Orange	0.6 ± 0.9	0.3
Apple	0 ± 0	0
Banana	0.8 ± 1.5	0.3
Kiwi	0 ± 0	0
Watermelon	0 ± 0	0
Mango	0 ± 0	0
Grape	0 ± 0	0
Strawberry	0 ± 0	0
Blueberry	0 ± 0	0
**Nuts and seeds**	**0.3 ± 0.9**	**0**
Almond	0.1 ± 0.4	0
Pistachio	0.1 ± 0.4	0
Peanut	0.1 ± 0.3	0

Table S1. Continued.

Food item	Mean ± SD (μg)	Median (μg)
**Poultry**	**0.4 ± 1.0**	**0.2**
Pork	0.2 ± 0.2	0.1
Beef	0 ± 0.1	0
Lamb	0.1 ± 2.2	0
Sausage and ham	0.1 ± 3.2	0.9
**Livestock**	**3.0 ± 4.6**	**1.4**
Chicken	2.6 ± 0.1	0.1
Duck	0.3 ± 0.9	0
**Milk and dairy products**	**16.0 ± 18.1**	**10.4**
Milk	10.8 ± 13.3	6.5
Cheese	0.9 ± 1.6	0.4
Yogurt	4.3 ± 9.0	0
**Eggs**	**12.3 ± 13.1**	**7.6**
Egg	11.6 ± 12.1	7.5
Preserved egg	0.7 ± 3.2	0
**Seafood**	**14.1 ± 24.5**	**8.3**
Sea-water fish	3.9 ± 9.5	0.6
Fresh-water fish	0.3 ± 0.6	0.1
Sashimi	0.9 ± 1.9	0.3
Fish ball	2.2 ± 5.0	1.1
Shrimp	1.0 ± 2.1	0.3
Crab	2.9 ± 7.5	0
Shellfish	2.6 ± 13.0	0.8
Squid	0.4 ± 1.2	0
**Other common foods and drinks**	**22.9 ± 52.1**	**8.9**
Egg waffle	1.1 ± 3.0	0.5
Cake	1.5 ± 2.2	0.8
French fries	0.7 ± 1.7	0.3
Chips	0.2 ± 0.5	0
Seaweed snack	4.9 ± 19.5	0
Kelp soup	10.1 ± 44.0	0
Black sesame soup	0 ± 0	0
Soda drink	0.9 ± 1.7	0.4
Carton-pack beverage	0 ± 0	0
Bubble milk tea	2.6 ± 4.1	1.1
Juice	0.9 ± 2.2	0
